# The Unusual Dominance of the Yeast Genus *Glaciozyma* in the Deeper Layer in an Antarctic Permafrost Core (Adélie Cove, Northern Victoria Land) Is Driven by Elemental Composition

**DOI:** 10.3390/jof9040435

**Published:** 2023-04-03

**Authors:** Ciro Sannino, Luigimaria Borruso, Ambra Mezzasoma, Benedetta Turchetti, Stefano Ponti, Pietro Buzzini, Tanja Mimmo, Mauro Guglielmin

**Affiliations:** 1 Industrial Yeasts Collection DBVPG, Department of Agricultural, Food and Environmental Sciences, University of Perugia, 06121 Perugia, Italy; 2 Faculty of Science and Technology, Free University of Bozen-Bolzano, 39100 Bozen-Bolzano, Italy; 3 Department of Theoretical and Applied Sciences, Insubria University, 21100 Varese, Italy

**Keywords:** Antarctica, abiotic parameters, chemical–physical parameters, fungal community, metabarcoding, rock glaciers, ice core

## Abstract

Rock glaciers are relatively common in Antarctic permafrost areas and could be considered postglacial cryogenic landforms. Although the extensive presence of rock glaciers, their chemical–physical and biotic composition remain scarce. Chemical–physical parameters and fungal community (by sequencing the ITS2 rDNA, Illumina MiSeq) parameters of a permafrost core were studied. The permafrost core, reaching a depth of 6.10 m, was divided into five units based on ice content. The five units (U1–U5) of the permafrost core exhibited several significant (*p* < 0.05) differences in terms of chemical and physical characteristics, and significant (*p* < 0.05) higher values of Ca, K, Li, Mg, Mn, S, and Sr were found in U5. Yeasts dominated on filamentous fungi in all the units of the permafrost core; additionally, Ascomycota was the prevalent phylum among filamentous forms, while Basidiomycota was the dominant phylum among yeasts. Surprisingly, in U5 the amplicon sequence variants (ASVs) assigned to the yeast genus *Glaciozyma* represented about two-thirds of the total reads. This result may be considered extremely rare in Antarctic yeast diversity, especially in permafrost habitats. Based on of the chemical–physical composition of the units, the dominance of *Glaciozyma* in the deepest unit was correlated with the elemental composition of the core.

## 1. Introduction

Antarctica includes ecosystems generally characterized by temperatures below 5° C. Low liquid water and nutrients, high UV incidence, and recurrent freezing cycles affected the diversity of psychrophilic microbial communities, which have developed a high level of adaptations to such extreme conditions [1,2,3,4]. Fungi (both in the yeast and filamentous life forms) are among the dominant decomposers degrading organic macromolecules at low (even sub-zero) temperatures, thus contributing to biogeochemical cycles in these ecosystems [5,6,7,8,9,10,11]. Therefore, understanding the environmental parameters driving fungal diversity is crucial for elucidating their ecology in Antarctic ecosystems. In recent decades, several studies on fungal diversity associated with Antarctic permafrost found the presence of a number of psychrophilic microbial taxa [12,13,14,15,16,17]. Many fungal genera (i.e., *Dioszegia* and *Naganishia* among yeasts and *Cladosporium*, *Curvularia*, *Leotiomycetes*, *Mortierella*, *Penicillium*, and *Pseudogymnoascus* among filamentous fungi) exhibited a strong prevalence, while others (i.e., *Candida*, *Filobasidium*, *Glaciozyma* and *Pichia* among yeasts and *Leptosphaeria* and *Rhizopus* among filamentous fungi) have always been observed less frequently, so these have long been considered minor taxa of little ecological significance. Among them, the yeast genus *Glaciozyma* has been constantly reported as one of the minor genera found within the Antarctic fungal communities [14,17,18]. Rock glaciers in Antarctic permafrost areas, such as Northern Victoria Land, are quite common and could be considered postglacial cryogenic landforms [19]. Rock glaciers are masses of coarse angular debris that display steep fronts and a system of transverse surface ridges and furrows, indicating a downward flow movement. They are widespread geomorphological features throughout the cryotic areas of both the Southern and Northern Hemispheres, including Antarctica [19] and references therein. Although the extensive presence and importance of rock glaciers, information on their chemical–physical (abiotic) and biotic composition remains scarce. Specifically, there is no information about the influence of chemical–physical drivers on fungal diversity associated with the permafrost of Antarctic rock glaciers.

With this regard, the challenges in this work were: (i) what is the structure of the fungal community of the permafrost core of Antarctic rock glaciers? (ii) What chemical–physical factors are putatively driving fungal diversity herein found, including dominant fungal taxa? (iii) Could fungal diversity (including its interaction with chemical–physical parameters) be used as proxy to postulate hypotheses about the origin of the different permafrost layers?

## 2. Materials and Methods

### 2.1. Site Description and Samples Collection

**Figure 1:**
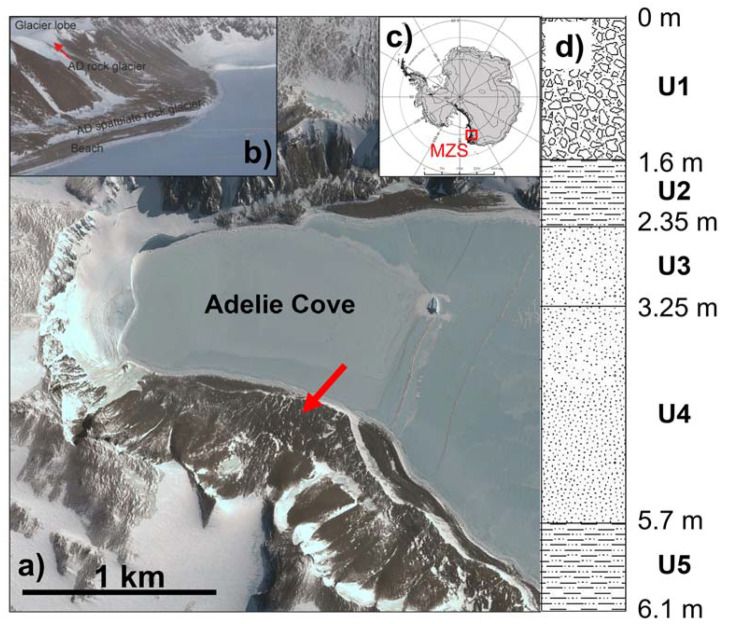
Location map. (**a**) satellite photographs (courtesy Google Earth^©^ DigitalGlobe 2016); (**b**) Adélie Cove South: from the sea to the upward margin, the raised beach, lower spatulate rock glacier, and the AD rock glacier can be seen; (**c**) map of Antarctica showing the location of the study area; (**d**) permafrost core stratigraphy.

Adélie Cove is located close to the Italian Antarctic Station “Mario Zucchelli” (74°42′ S–164°60′ E) in Northern Victoria Land, Continental Antarctica (Figure 1a–c). This area is characterized by large ice-free areas where the bedrock, composed mainly of Ordovician granite, is generally covered by discontinuous and thin tills [19]. The studied permafrost core was drilled in 2003 on the rock glacier located on the southern side of Adélie Cove and stored at −20 °C until analysis. This rock glacier is tongue-shaped, 510 m long, and 280 m wide, and develops between 255 and 50 m a.s.l [19].

The permafrost core, reaching a depth of 6.10 m, was divided into five units (U) based on ice content according to the results reported by [19] (Figure 1d). A different number of samples/units used for physical chemical and microbiological studies was used; it was proportional to the length and the content and distribution of ice along with the depth of the different units. In detail: (i) U1: from 0 to 1.6 m depth (number of samples = 6), characterized by low ice content occurring as interstitial ice; (ii) U2: from 1.6 to 2.35 m (number of samples = 5), exhibiting an ice content composition highly variable with depth and including both centimetric ice lenses and interstitial ice; (iii) U3: from 2.35 to 3.25 m (number of samples = 4); and U4: from 3.25 to 5.70 m (number of samples = 6), both characterized by a massive ice body (average ice content of approximately 85%). In U3, the ice was relatively clean with the scarce presence of appreciable sediment layers, except for a 5 cm-thick sandy layer at a depth of 2.85 m. On the other hand, in U4, a thicker sand layer was observed between 3.25 and 3.40 m, a 5 cm thick layer of debris at 4.55 m, and—below the ice—several millimetric silty–sandy layers; U5: from 5.7 to 6.10 m (number of samples = 3), characterized by the presence of both centimetric ice lenses and interstitial ice highly variable with depth.

### 2.2. Chemical and Physical Analyses

Permafrost cores were surface-decontaminated in the laboratory according to Rogers et al. [20] to exclude the presence of external microorganisms on the sample surfaces introduced during drilling procedures. Water content (%) was calculated using weight loss by oven-drying the samples at 105 °C until a constant weight was reached [21], and pHH_2_O was determined on an oven-dried fine soil sample using a 1:1 soil:water ratio [22]. Electrical conductivity (µS/cm) for each sample was measured using an electrical conductivity meter (Hanna Instruments-HI 98360) with an accuracy of ±0.5%.

Total organic Carbon (TOC, g/Kg), total organic nitrogen (TON, mg/Kg), and total organic phosphorous (TOP, mg/Kg) were determined using standard methods, as reported by Mudroch et al. [23]. After filtration, 1:10 water extracts were analyzed (Whatman 41) via inductively coupled plasma–optic emission spectroscopy (ICP-OES, Arcos Ametek Spectro, Weiterstadt, Germany) for their elemental composition.

### 2.3. DNA Extraction, Library Preparation, and Sequencing

After decontamination and the thawing process described by Rogers et al. [20], total DNA from samples containing more than 10% of solid fraction was extracted from 0.25 g (wet weight) of each sample using a Power Soil DNA Isolation Kit (Qiagen, Hilden, Germany). All the samples with a solid fraction of less than 10% were aseptically filtered to collect the biomass present on one sterile cellulose acetate filter (cut-off = pore size 0.2 μm Sartorius Stedim, Biotech, Gottingen, Germany), and total DNA was extracted using a Power Water DNA Isolation Kit. Two separate extractions of samples with low DNA concentration were pooled to reach the final concentration of 1 ng/μL, assessed with a QuBit 3.0 Fluorometer Assay (Life Technologies Corporation, Carlsbad, CA, USA).

Fungal internal transcribed spacer region 2 (ITS2) was amplified using IlluAdp_ITS31_NeXTf 50-CATCGATGAAGAACGCAG-30 and IlluAdp_ITS4_NeXTr50-TCCTCCGCTTATTGATATGC-30 [24]. The PCR products followed the standard protocols of the Company Biofab research s.r.l. (Rome, Italy). Index Nextera XT were added to PCR products by the company. The sequencing platform used was MiSeq Sequencing reporting as raw sequences 300 × 2 cycles Paired End sequences.

### 2.4. Bioinformatics and Statistical Analysis

Raw sequences were processed using the Amplicon ToolKit (AMPtk v.1.5.2) for NGS data (formally UFITS) v.1.4.1 [25]. Primers and barcodes were removed from the sequences and reads were trimmed to 250 bp. Reads shorter than 100 bp and chimera were removed by USEARCH [26]. Merged raw reads were analyzed in AMPtk using the “dada2” algorithm to generate representative amplicon sequence variants (ASVs) during the phase AMPTK clustering. The determination of ASVs was preferred to operational taxonomic units (OTUs) because this approach can provide a more precise identification of microbial communities and can provide a more detailed picture of the diversity within a given sample, consistent with current literature [27,28].

Singletons and rare taxa (<5 reads) were removed as likely false positives due to sequencing errors [29]. Taxonomic assignment was based on the results of global alignment (USEARCH/VSEARCH), UTAX, and SINTAX [26]. All sequences have been submitted to the European Nucleotide Archive (EMBL–EBI) under the accession number PRJEB46030.

Statistical analyses were performed using statistical multi-packages of R software version 4.0.3 [30]. Alpha- and beta-diversity analyses were performed on the rarefied SV table with a sampling depth of 79,504 reads. Differences in fungal alpha-diversity, including Richness and Shannon-H indexes, were tested via Kruskal–Wallis followed by a post hoc pairwise multiple comparison procedure (Wilcoxon test), using the whole dataset.

Fungal beta-diversity was calculated via Bray–Curtis distance, and differences were tested using the permutational multivariate analysis of variance (PERMANOVA) via the function “adonis” implemented in the vegan package in R, using the whole dataset. Non-metric multidimensional scaling (NMDS) was conducted, and the envfit function was used to assess the impacts of chemical–physical parameters on fungal community composition. The ordisurf function was used to plot the depth. Only the significant (*p* < 0.05) vectors were fitted to the NMDS ordination.

Co-occurrences among chemical–physical parameters and fungal genera (with a relative abundance > 2%) were calculated based on Pearson correlation coefficient and visualized by using Cytoscape [31]. Only significant correlations reporting a *p*-value < 0.05 were considered. The nodes in the reconstructed network represent the chemical–physical parameters and the fungal genera, whereas the green edges correspond to a positive and significant correlation between nodes; red edges correspond to a negative and significant correlation between nodes. The online tool (www.interactivenn.net) was used to sketch the Venn diagram [32].

## 3. Results

### 3.1. Chemical and Physical Analysis

The five units (U1–U5) of the permafrost core exhibited several significant (*p* < 0.05) differences in terms of chemical and physical parameters (Supplementary Table S1). The highest (*p* < 0.05) value of TOC was found in U1. On the contrary, the higher (*p* < 0.05) values of TON were found in U1 and U2 in comparison with U4 and U5 (Supplementary Table S1). Overall, the water content (Wt) varied (*p* < 0.05) along the whole depth of the core, from the lowest value observed in U1 to the highest found in U3 and U4. Likewise, electrical conductivity (EC) significantly (*p* < 0.05) differed among the five units, with a general trend of increasing with depth. On the other hand, pH showed the most alkaline (*p* < 0.05) value in U1 (Supplementary Table S1).

Considering the elemental composition, significant (*p* < 0.05) higher values of Fe and Ti were found in U1, while significant (*p* < 0.05) higher values of Ca, K, Li, Mg, Mn, S, and Sr were found in U5. Other elements (Cl, Mo, Na and P) exhibited a more fluctuating trend with depth (Table 1).

### 3.2. Taxonomic Structure of Fungal Communities in Permafrost Core

**Figure 2:**
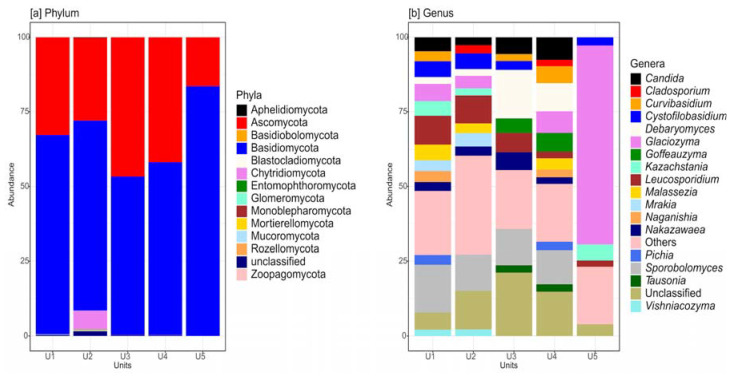
Fungal diversity (considering filamentous fungi, yeasts, and unclassified taxa) found in the five units of the permafrost core of Adelie Cove rock glacier (U1, U2, U3, U4, and U5). Relative abundance of ASVs distribution at phylum level (**a**); relative abundance of ASVs distribution at genus level (**b**).

After bioinformatic analyses, a total of 2,897,213 fungal reads, grouped into 2783 ASVs, were found. A high percentage of unclassified reads was found. In detail from phylum level to genera level the average percentage of unclassified reads was 0.5% (phylum level, range 0.1–1.6%), 1.4% (class level, range 0.3–5.5%), 1.6% (order level, range 0.3–6%), 8.2% (family level, range 3.8–12.3%), and 11.7% (genera level, range 4–21.2%) (Figure 2 and Supplementary Figures S1–S3).

**Figure 3:**
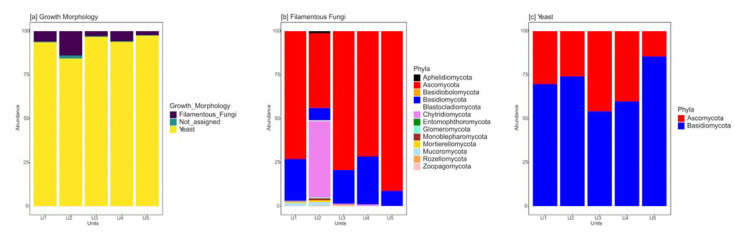
Fungal diversity differently found in the five units of the permafrost core of Adelie Cove rock glacier (U1, U2, U3, U4, and U5). (**a**) Relative abundance of ASVs assigned to yeasts and filamentous fungi and their distribution among these two life forms; (**b**) relative abundance of ASVs assigned only to filamentous fungi phyla; and (**c**) relative abundance of ASVs assigned only to yeast phyla.

The fungal community was examined by dividing ASVs into yeasts and filamentous fungal taxa. Overall, yeasts dominated on filamentous fungi: the ASVs assigned to yeast life forms reported abundances from 84.4 to 97.4% of the total, while ASVs assigned to filamentous life forms accounted for percentages ranging from 2.3 to 13.9% (Figure 3a). Ascomycota was the prevalent phylum among filamentous forms (Figure 3b), while Basidiomycota was the dominant phylum among yeast forms in all units (U1–U5) of the permafrost core (Figure 3c).

Considering the whole fungal community (yeasts + filamentous fungi), ASVs assigned to Ascomycota (abundance from 16.4 to 46.7%) and Basidiomycota (abundance from 52.9 to 83.5%) represented almost all of the diversity observed, with the sole exception of ASVs assigned to Chytridiomycota, which accounted for 6% of total in U2 (Figure 2a). At the genus level, a clear dichotomic trend was found along the core depth, with the deepest layer (U5) greatly differing from those above (U1–U4). *Debaryomyces*, *Leucosporidium* and *Sporobolomyces* were the most abundant genera in U1, U2, U3, and U4. In particular, *Sporobolomyces* and *Leucosporidium* dominated in both U1 and U2 (*Sporobolomyces* 15.9 and 12.1%, *Leucosporidium* 9.6 and 9.3%, respectively), *Debaryomyces* (16.3%) and *Sporobolomyces* (12.1%) in U3, while *Leucosporidium* (11.3%) and *Debaryomyces* (9.5%) were the most abundant genera in U4. Surprisingly, in U5, the ASVs assigned to the genus *Glaciozyma* represented about two thirds of the total reads, while in U1–U4 the ASVs assigned to the same genus always accounted to value below 5% (Figure 2b). The Venn diagram indicates that 21.8% of ASVs found are common to the five units (U1–U5) of the permafrost core, whereas 8% of ASVs were found exclusively in U1, 9.2% in U2, 3.8% in U3, 7.7% in U4, and only 2.9% in U5 (Supplementary Figure S4).

**Figure 4:**
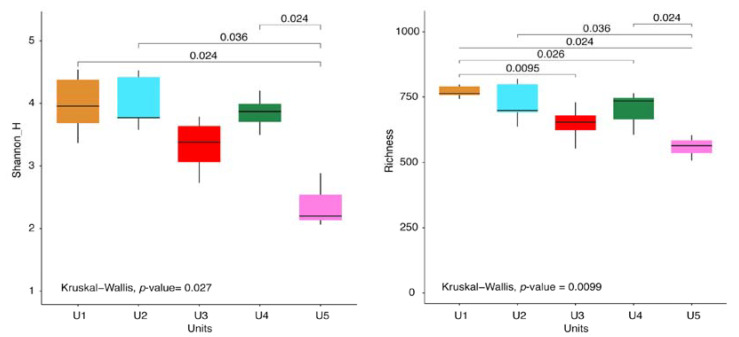
Alpha-diversity indices (Shannon-H left panel, richness right panel) of the five units of the permafrost core of Adélie Cove rock glacier (considering filamentous fungi, yeasts, and unclassified taxa). Significant (*p* < 0.05) differences are highlighted by connectors.

Richness and Shannon-H indexes revealed that U5 exhibited the lowest diversity (*p* < 0.05) (Figure 4).

**Figure 5:**
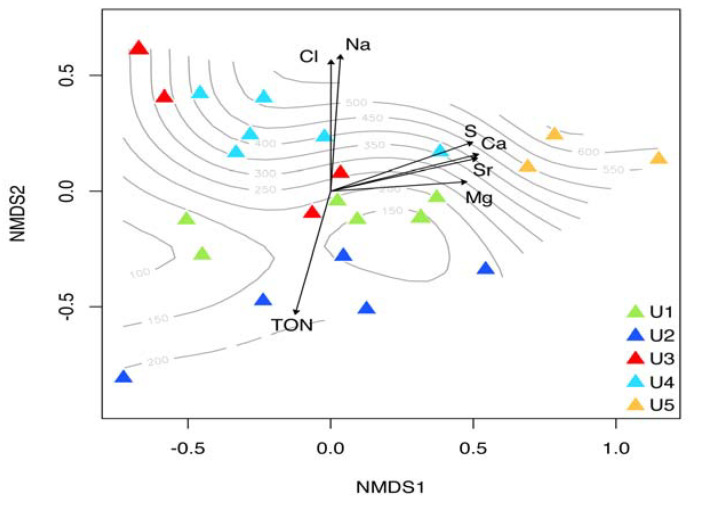
Non-metric multidimensional scaling (NMDS) plot based on Bray-Curtis distance of fungal communities found in the five permafrost units (U1, U2, U3, U4, and U5) (considering filamentous fungi, yeasts, and unclassified taxa). Dashed lines represent the gradient of depth. The envfit function was used to show the chemical and physical parameters affecting fungal communities. Only the significant (*p* < 0.05) vectors were fitted to the NMDS ordination.

The beta-diversity analysis visualized via NMDS and tested via Permanova reported a trend where significant (*p* < 0.01) differences were found among U1–U5 core layers (Table 1). The parameters influencing the fungal beta-diversity on NMDS1 were S, Ca, Sr, and Mg. On the other hand, Cl, Na, and TON resulted in driving the distribution of the samples on NMDS2 (Figure 5 and Table 2).

### 3.3. Pearson Correlation and Influence of Chemical–Physical Parameters

**Figure 6:**
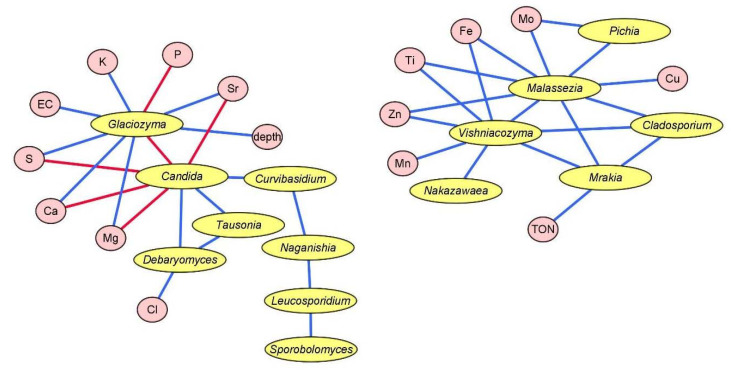
Pearson correlations between chemical and physical parameters (pink circles) and fungal taxa (abundance > 2%) (yellow circles) found in the five units of permafrost core of Adélie Cove rock glacier. Blue lines show positive significant (*p* < 0.05) correlations. Red lines show negative significant (*p* < 0.05) correlations. EC, electrical conductivity; TOC, total organic carbon; TON, total organic nitrogen.

The Pearson correlation coefficient was calculated to establish the putative interactions between fungal ASVs (assigned at the genus level, abundance > 2%) and chemical-physical parameters. Considering the Pearson correlation coefficient among fungal genera, a high number of significant (*p* < 0.05) positive correlations was found. Overall, correlations involved only yeasts vs. yeasts and yeasts vs. chemical–physical parameter interactions (Figure 6 and Supplementary Table S2). In particular, the yeast genera reporting the highest number of positive correlations were (i) *Candida* vs. *Debaryomyces*, *Tausonia*, and *Curvibasidium*; (ii) *Malassezia* vs. *Cladosporium* and *Pichia*; (iii) *Mrakia* vs. *Malassezia*, *Cladosporium*, and *Vishniacozyma*; and (iv) *Vishniacozyma* vs. *Cladosporium*, *Malassezia*, and *Nakazawaea*. Interestingly, *Glaciozyma* vs. *Candida* was the unique significant (*p* < 0.05) negative correlation between fungi found at the genus level (Figure 6 and Supplementary Table S2).

Considering the correlations between chemical–physical parameters and fungal ASVs, a number of significant (*p* < 0.05) positive correlations were found: (i) *Glaciozyma* vs. depth, EC, Ca, K, Mg, S, and Sr; (ii) *Malassezia* vs. Fe, Mo, Ti, and Zn; (iii) *Vishniacozyma* vs. Fe, Mn, Ti, and Zn; (iv) *Mrakia* vs. TON; (v) *Pichia* vs. Mo; and (vi) *Debaryomyces* vs. Cl (Figure 6 and Supplementary Table S2). On the contrary, only a few significant (*p* < 0.05) negative correlations were found: (i) *Glaciozyma* vs. P and (ii) *Candida* vs. Ca, Mg, S, and Sr (Figure 6 and Supplementary Table S2).

## 4. Discussion

Cryogenic environments of Earth, including Antarctica, harbor a wide diversity of psychrophilic and psychrotolerant microorganisms [33,34] and reference therein. Despite the number of studies on microbial communities sharing Antarctic habitats [17,35,36,37,38], only a few mycological studies which have taken selected habitats into consideration have been performed to date. Among them, Antarctic permafrost may still be considered partially unexplored from a mycological point of view [18].

A succession of heterogeneous habitats was found along the depth in the studied permafrost core; this heterogeneity significantly affected the structure of fungal communities, in agreement with previous research reporting that depth-related variations of microbial diversity in both cold soils and permafrost cores [39,40,41].

The general dominance of yeast on filamentous life forms is consistent with previous studies carried out in cold environments worldwide, including Antarctica, suggesting that the unicellular lifestyle could be better adapted to cold than hyphal one [10,17,34,35,42,43]. All genera found as dominant in the present study (i.e., *Debaryomyces*, *Glaciozyma*, *Leucosporidium*, and *Sporobolomyces*) exhibit a psychrophilic or psychrotolerant habit [34,35,43]. The high abundance of yeasts belonging to Basidiomycota may be attributed to their apparent higher ability to express some adaptive physiological modifications, namely the production of polysaccharide capsules, the preservation of cell fluidity at sub-zero temperatures (by increasing the proportion of unsaturated fatty acids in cell membranes), and the ability to release cold-active enzymes [8,34,44,45,46,47,48].

The permafrost core was preliminarily divided into five units (U1–U5) according to the typology and the amount of ice, as reported by Guglielmin et al. [19]. It is remarkable as the fungal diversity confirms the geological interpretation of the geophysical results of Guglielmin et al. [19]; indeed, U1 and U2 were suggested as interpretable as permafrost with an active layer of 0.65 m, and U3 and U4 were separated from the upper ones by a reflector composed of relict glacier ice; U5 was not clearly detected by the GPR, and the borehole was too thin to determine if it could be a part of an older sequence of sediments developed in cryotic ice-free areas or if it could be the frozen till of an older relict glacier.

The fungal diversity found in U5 is to be considered definitely uncommon due to the substantial prevalence of ASVs ascribed to the genus *Glaciozyma*, representing about two thirds of the total reads. This result may be considered extremely rare in Antarctic yeast diversity, especially in permafrost habitats. With the sole exception of a study carried out on soils of the McMurdo Dry Valleys, where ASVs belonging to this genus dominated the fungal diversity of Hjorth Hill (HH) site [49], no references so far reported the prevalence of *Glaciozyma* in cold ecosystems worldwide. Interestingly, while in the study of Thompson et al. [49] the same genus was found close to the upper part of the soil in a site rich in organic mat (mosses), so under conditions truly unrelated to glacial ice, in the present study, *Glaciozyma* was particularly abundant in completely different environmental conditions. Therefore, U5 could be considered a hotspot for this yeast genus.

The yeast genus *Glaciozyma* was proposed for the first time by Turchetti et al. [50], who described two new species (i.e., *G. martinii* and *G. watsonii*) and reclassified *Leucosporidium antarcticum* as *G*. *antarctica*. Due to its psychrophilic habit, *Glaciozyma* was found, but as minor fungal taxon, in both Arctic [51,52,53,54,55] and Antarctic sites [49,54,56,57]. The genome of *G. antarctica* has been recently sequenced and studied [46], reporting systems of psychrophilic responses apparently allowing yeast growth at very low (or even sub-zero) temperatures. For instance, a significant induction of antifreeze proteins (AFPs) and fatty acid desaturases (FADs) was observed after exposure of *G. antarctica* to growth temperature ranging from 0 to 5 °C; a combined effect of upregulation of FAD gene and an over-regulation of AFP has been observed at 0 °C. This combined effect is realized to increase cell membrane fluidity and avoid ice crystallization inside the cells under cold conditions [58]. A more recent study reported that *G. antarctica* cells exposed to −12 °C could up-regulate the gene coding for trehalose 6-phosphate phosphatase, highlighting the importance of trehalose accumulation to prevent ice formation inside the cell [59].

Only some speculation can be made about the origin of the accumulation of *Glaciozyma* in U5, which can be considered uncommon in this environment. A possible hypothesis could be the presence of such older layer U5 with a well-established biological diversity (where *Glaciozyma* was the dominant taxon) and the subsequent burial of this layer by the glacier (U3 and U4) and its morainic coverage (U1 and U2) in which a different fungal microbiota may have developed. It is also possible to speculate that this relict glacier could be a possible remnant of the Ross Sea Ice Shelf that until almost 11,000 years ago covered this sector of Northern Victoria Land [60,61].

The role of chemical–physical parameters could be considered crucial for finding the rationale justifying the accumulation of *Glaciozyma* in the deeper layer, U5. In particular some essential elements involved in yeast cell physiology [62,63], and biochemical processes as enzymatic cofactors [64] could be relevant. Indeed, the abundance of *Glaciozyma* resulted positively (*p* < 0.05) correlated with depth, EC, Ca, K, Mg, S, and Sr. Previous studies suggested that some organisms correlate with the element content associated with a given habitat in dependence on their bioavailability [65,66]. Bioinformatic approaches investigated the presence of putative metal-binding proteins (containing specific metal-binding active sites) produced by some cold-adapted fungi [67,68]. Metal-binding proteins and metal-binding domains were studied in *Glaciozyma* spp. to depict its mechanism of metal acquisition to survive under freezing temperatures. Foong et al. [69] found that a quarter of *G. antarctica* proteome is metal-enriched, with a prevalence of Zn. Interestingly, the same study hypothesized that *G. antarctica* exhibited a non-specific trend of metal usage resembling that found in some non-psychrophilic yeasts, including the enrichment of proteome with some essential nutrients, such as Ca and Mg, which was found in this study positively correlated with the abundance of *Glaciozyma* [69]. Li et al. [70] reported the importance of Ca in the composition of antifreeze proteins produced by Atlantic herring fish (Clupea harengus), which play a fundamental role in preventing the formation of ice crystals. Moreover, a study on a 3D model of a cold-active α-amylase (AmyPI12) produced by *G. antarctica* revealed the presence of binding sites for Ca and Na [71]. The effects of various elements on the activity of some cold-active hydrolases released by *G. antarctica* PI12, i.e., an esterase-like protein (GaDlh) and a hormone-sensitive lipase (HSL)-like esterase (GlaEst12) were recently tested [72,73]. The activity of GaDlh was inhibited in the presence of Zn and Cu2, while the presence of Mg and Ca determined only partial inhibition, respectively [72]. On the other hand, Na, K, Ca, and Mn enhanced the esterase activity of GlaEst12, while Mg, Ni, and Cu acted in a reverse manner by inhibiting the GlaEst12 activity [73].

## Figures and Tables

**Table 1:** Estimation of fungal beta-diversity by multivariate Permutation test for adonis (n. permutation 999) under reduced model of the different permafrost units (U).

	Df	Sum of Sqs	R2	F Value	*p* Value
Units	4	2.26	0.37	2.81	0.001
Residual	19	3.82	0.62		
Total	23	6.08	1.00		

**Table 2:** Results of envfit function showing the relationship of chemical/physical parameters with Non-metric multidimensional scaling (NMDS) ordination of the permafrost-associated fungal communities. TOC, total organic carbon; TON, total organic nitrogen; EC, electrical conductivity. Significant (*p* < 0.05) *p* values are shown in bold.

Factors	NMDS1	NMDS2	r2	Pr (>r)
Depth	0.40638	0.9137	0.4361	**0.003996**
TOC	0.00772	−0.99997	0.1003	0.338661
TON	−0.22719	−0.97385	0.524	**0.000999**
Water	−0.08076	0.99673	0.1688	0.144855
EC	0.63623	0.7715	0.234	**0.042957**
pH	−0.22593	−0.97414	0.0241	0.796204
Ca	0.95569	0.29437	0.5153	**0.003996**
Cl	0.00254	1	0.5632	**0.000999**
Cu	−0.99938	−0.03532	0.091	0.35964
Fe	−0.45623	−0.88986	0.0633	0.491508
K	0.80512	0.59311	0.2392	0.061938
Li	0.50517	−0.86302	0.0699	0.492507
Mg	0.99634	0.08553	0.4009	**0.007992**
Mn	0.21197	−0.97728	0.0724	0.444555
Mo	−0.09002	−0.99594	0.2351	0.053946
Na	0.05836	0.9983	0.611	**0.000999**
P	−0.81812	−0.57504	0.107	0.283716
S	0.91985	0.39227	0.5127	**0.003996**
Sr	0.96376	0.26678	0.4985	**0.003996**
Ti	−0.46031	−0.88776	0.0645	0.482517
Zn	−0.25115	−0.96795	0.052	0.565435

## Data Availability

The data presented in this study are openly available in the European Nucleotide Archive (EMBL–EBI) under the accession number PRJEB46030.

## Supplementary Materials

The following supporting information can be downloaded at: https://www.mdpi.com/article/10.3390/jof9040435/s1, Supplementary Figure S1. Fungal diversity (considering both filamentous fungi and yeasts) found in the permafrost core of Adelie Cove rock glacier. Relative abundance of ASVs distribution at class level. Supplementary Figure S2. Fungal diversity (considering both filamentous fungi and yeasts) found in the permafrost core of Adelie Cove rock glacier. Relative abundance of ASVs distribution at Order level. Supplementary Figure S3. Fungal diversity (considering both filamentous fungi and yeasts) found in the permafrost core of Adelie Cove rock glacier. Relative abundance of ASVs distribution at Family level. Supplementary Figure S4: Venn diagram showing the number of fungal amplicon sequence variants (ASVs) in permafrost core (units U1–U5) of Adélie Cove rock glacier. Description of U1–U5 is reported in the text; Table S1: Chemical and physical parameters of permafrost core (units U1–U5) of Adélie Cove rock glacier. Description of U1–U5 is reported in the text; Table S2: Significant (*p* < 0.05) correlations between chemical-physical parameters and fungal ASVs calculated by Pearson coefficient among fungal ASVs at genus level (abundance > 2%) and chemical and physical parameters and highlighted in the Pearson correlations analysis.
