# Supplementary material for: The Unusual Dominance of the Yeast Genus Glaciozyma in the Deeper Layer in an Antarctic Permafrost Core (Adélie Cove, Northern Victoria Land) Is Driven by Elemental Composition
Source: J Fungi (Basel). 2023 Apr 3;9(4):435. doi: 10.3390/jof9040435 (PMC10145851; doi:10.3390/jof9040435)
Supplement: Supplementary file 1 [file jof-09-00435-s001.zip › jof-2253338-supplementary.pdf]

# The Unusual Dominance of the Yeast Genus *Glaciozyma* in the Deeper Layer in an Antarctic Permafrost Core (Adélie Cove, Northern Victoria Land) Is Driven by Elemental Composition

**Ciro Sannino [1,†], Luigimaria Borruso [2,†], Ambra Mezzasoma [1], Benedetta Turchetti [1,*], Stefano Ponti [3], Pietro Buzzini [1], Tanja Mimmo [2] and Mauro Guglielmin [3]**

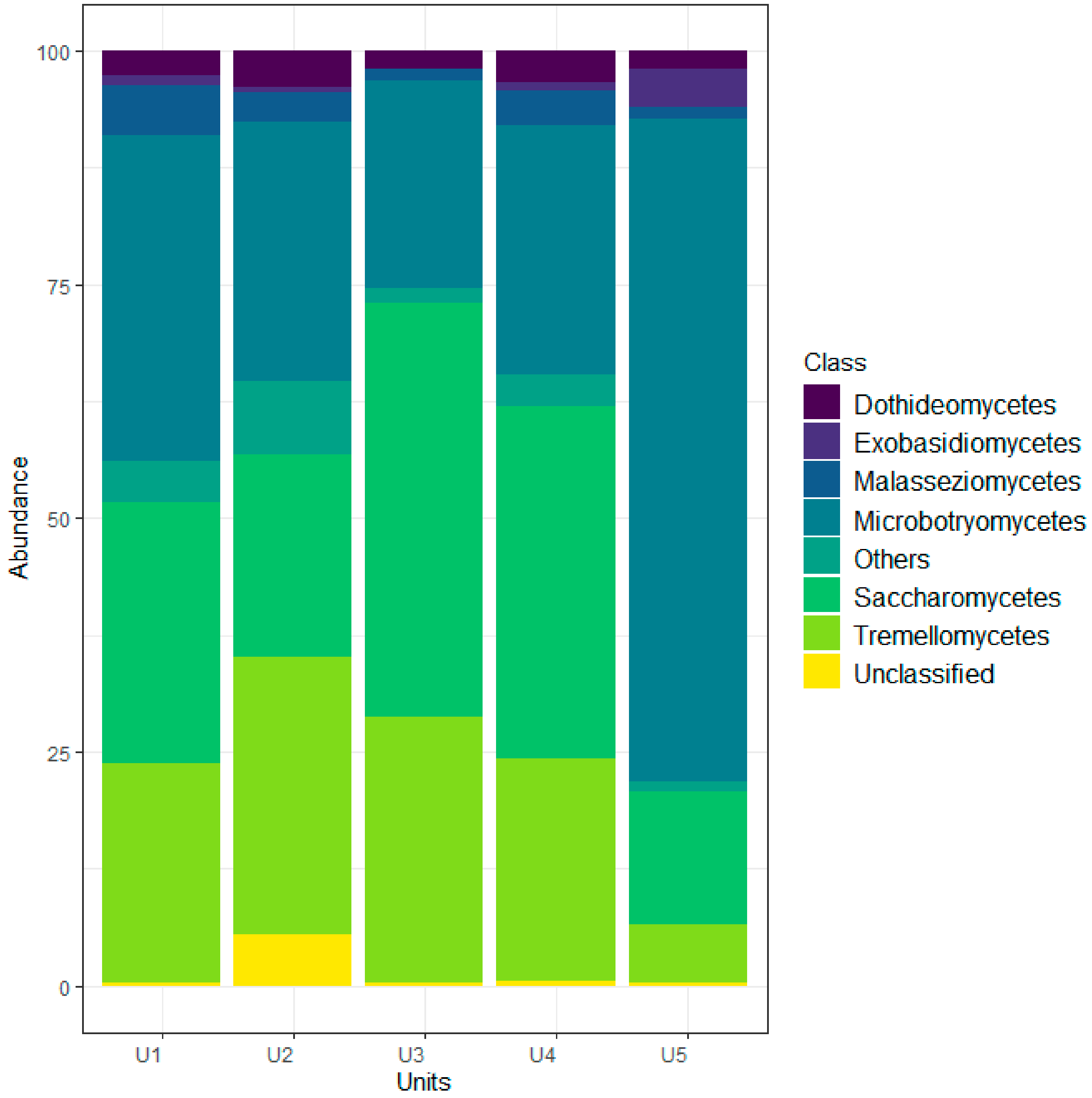


**Supplementary Figure S1.** Fungal diversity (considering both filamentous fungi and yeasts) found in the permafrost core of Adelie Cove rock glacier. Relative abundance of ASVs distribution at class level.

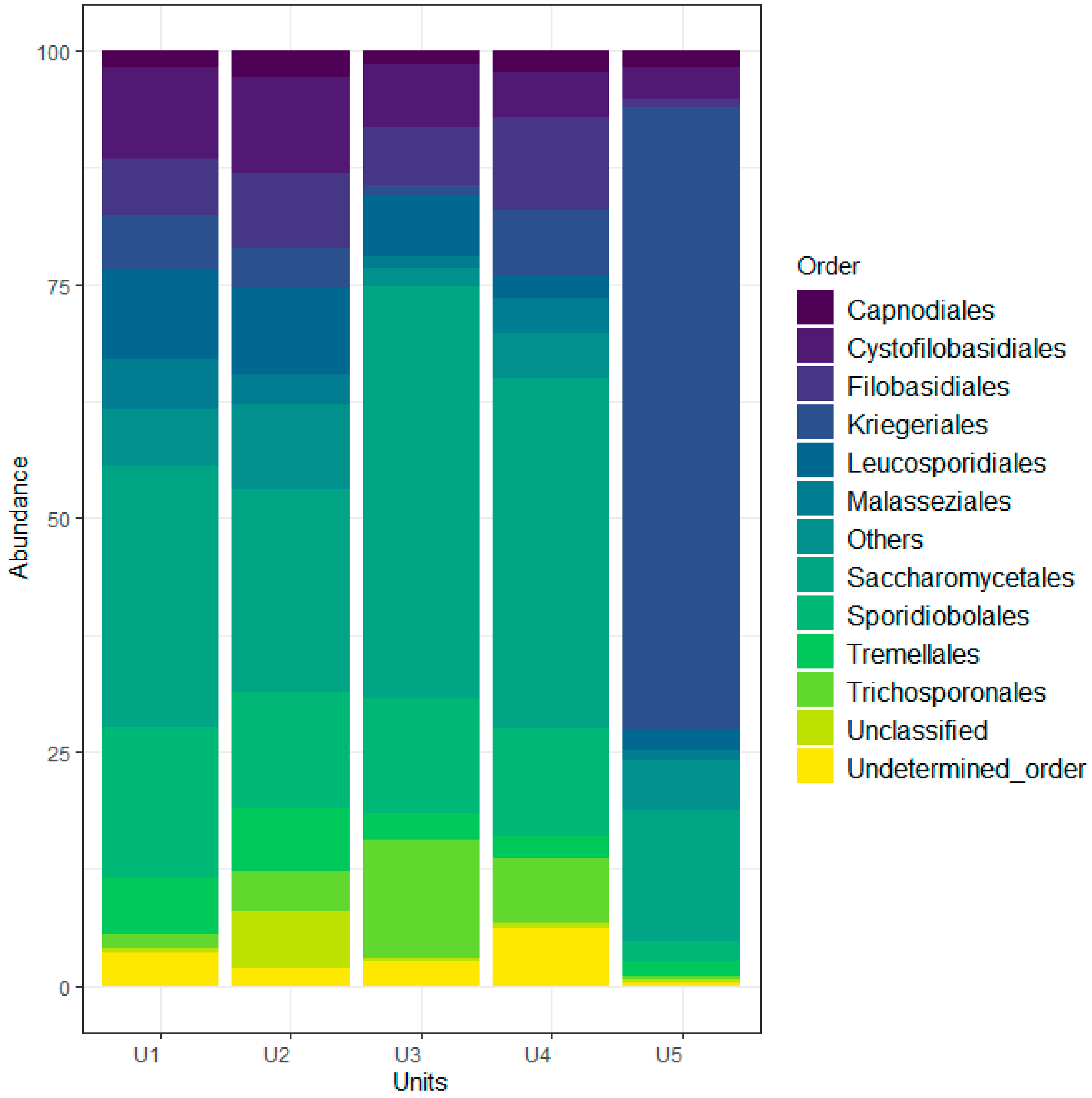


**Supplementary Figure S2.** Fungal diversity (considering both filamentous fungi and yeasts) found in the permafrost core of Adelie Cove rock glacier. Relative abundance of ASVs distribution at Order level.

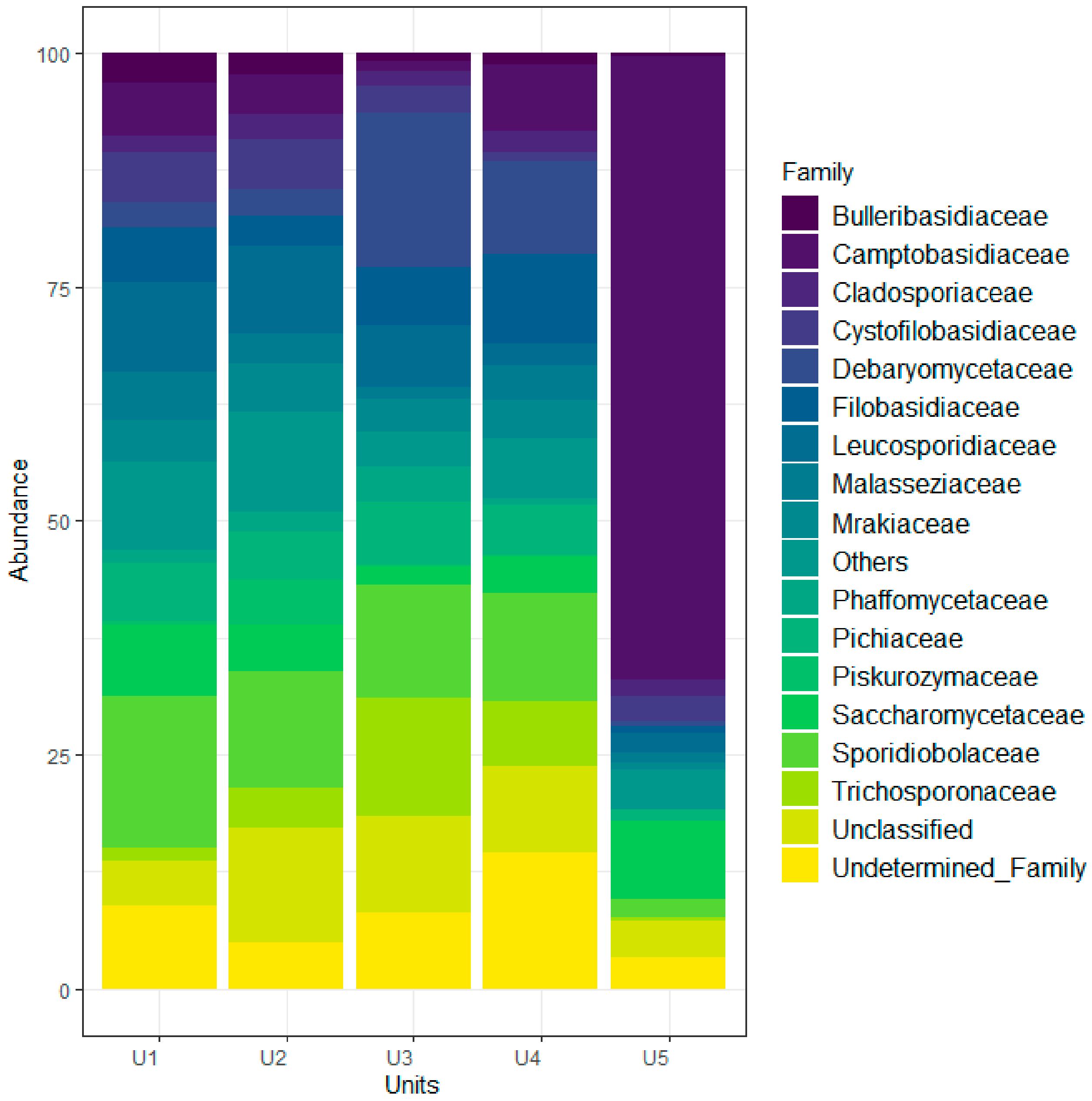


**Supplementary Figure S3.** Fungal diversity (considering both filamentous fungi and yeasts) found in the permafrost core of Adelie Cove rock glacier. Relative abundance of ASVs distribution at Family level.

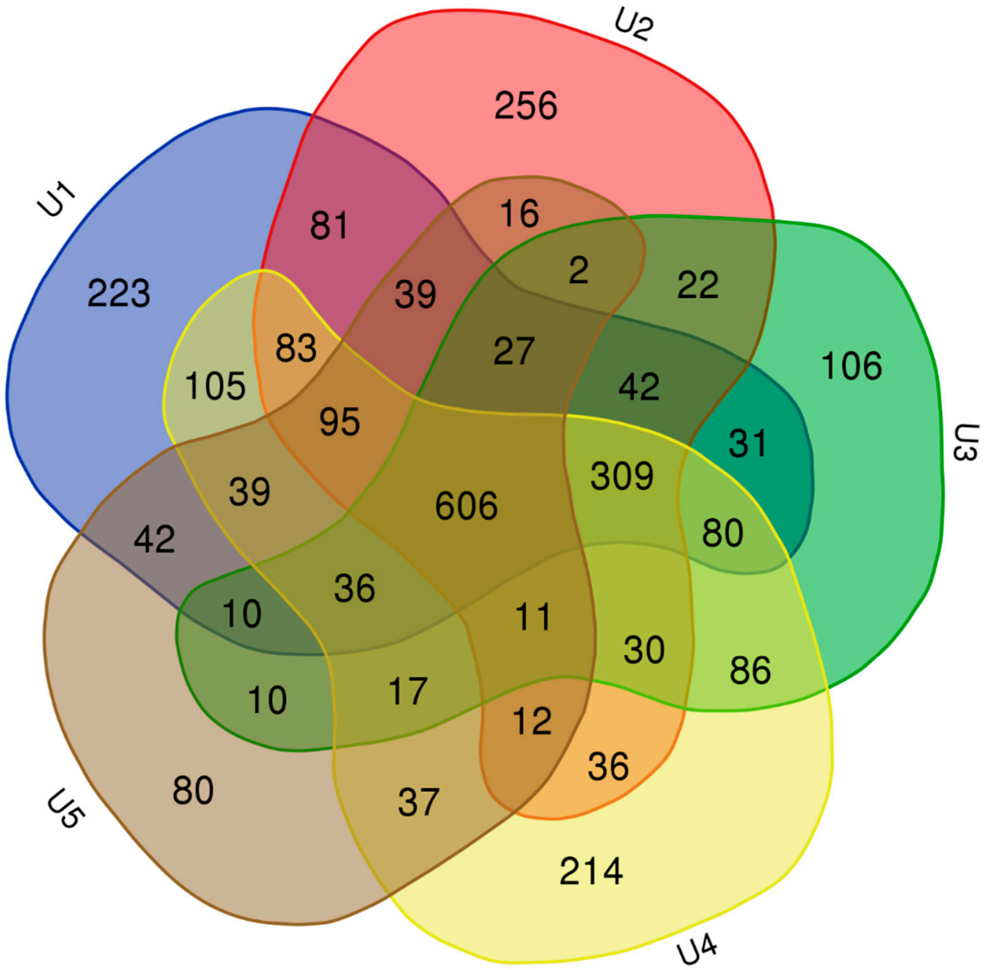


**Figure S4.** Venn diagram showing the number of fungal amplicon sequence variants (ASVs) in permafrost core (units U1-U5) of Adélie Cove rock glacier. Description of U1-U5 is reported in the text.

**Table S1.** Chemical and physical parameters of permafrost core (units U1-U5) of Adélie Cove rock glacier. Description of U1-U5 is reported in the text. TOC = Total Organic Carbon; TON = Total Organic Nitrogen; EC = Electrical Conductivity; Ca = calcium; Cl = chlorine; Cu = copper; Fe = iron; K = potassium; Li = lithium; Mg = magnesium; Mn = manganese; Mo = molybdenum; Na = sodium; P = phosphorous; S = sulphur; Sr = strontium; Ti = titanium; Zn = zinc. Significant ($p < 0.05$) p values are shown in bold. Superscript letters (calculated for each parameters) indicated significant ($p < 0.05$) differences by ANOVA and Tukey post-hoc multiple comparison.

| Units | depth (cm) | TOC (g/Kg) | TON (mg/Kg) | Water (%) | EC (µS/cm) | pH |
|---|---|---|---|---|---|---|
| U1 | 0-160 | 24.72±0.95$^{a}$ | 53.83±6.46$^{a}$ | 8.08±1.56$^{a}$ | 153.12±31.70$^{a}$ | 8.96±0.11$^{a}$ |
| U2 | 160-235 | 9.36±1.65$^{b}$ | 61.73±32.75$^{a}$ | 61.88±30.25$^{b}$ | 875.58±905.20$^{b}$ | 7.72±0.36$^{b}$ |
| U3 | 235-325 | 10.06±1.88$^{b}$ | 25.87±8.46$^{ac}$ | 80.75±30.02$^{c}$ | 471.89±1096.22$^{c}$ | 7.92±0.29$^{b}$ |
| U4 | 325-570 | 4.10±0.49$^{c}$ | 9.46±5.03$^{bc}$ | 83.73±7.74$^{c}$ | 1404.73±1172.50$^{d}$ | 7.75±0.35$^{b}$ |
| U5 | 570-610 | 8.60±0.86$^{b}$ | 8.40±3.18$^{bc}$ | 69.7±28.99$^{d}$ | 1735±1136.59$^{e}$ | 7.79±0.48$^{b}$ |

| Units | Na (mg/g) | Cl (mg/g) | Ca (mg/g) | K (mg/g) | Mg (mg/g) | P (mg/g) | S (mg/g) | Cu (µg/g) | Fe (µg/g) | Zn (µg/g) | Mn (µg/g) | Mo (µg/g) | Sr (µg/g) | Ti (µg/g) | Li (µg/g) |
|---|---|---|---|---|---|---|---|---|---|---|---|---|---|---|---|
| U1 | 0.65±0.38$^{a}$ | 0.92±0.53$^{a}$ | 0.06±0.02$^{a}$ | 0.10±0.02$^{a}$ | 0.03±0.01$^{a}$ | 0.002±0.00$^{a}$ | 0.03±0.01$^{a}$ | 0.31±0.13$^{a}$ | 23.17±17.61$^{a}$ | 0.25±0.13$^{a}$ | 0.45±0.27$^{ab}$ | 0.11±0.01$^{a}$ | 0.49±0.13$^{a}$ | 1.94±1.46$^{a}$ | 0.23±0.04$^{a}$ |
| U2 | 0.30±0.08$^{a}$ | 0.50±0.14$^{a}$ | 0.14±0.04$^{a}$ | 0.06±0.02$^{a}$ | 0.05±0.01$^{a}$ | 0.002±0.001$^{a}$ | 0.07±0.02$^{a}$ | 0.08±0.02$^{b}$ | 1.63±0.38$^{b}$ | 0.06±0.02$^{b}$ | 0.29±0.08$^{a}$ | 0.16±0.04$^{b}$ | 0.78±0.21$^{ac}$ | 0.13±0.03$^{b}$ | 0.12±0.03$^{b}$ |
| U3 | 8.46±0.08$^{b}$ | 10.99±0.21$^{b}$ | 0.23±0.01$^{a}$ | 0.25±0.01$^{b}$ | 0.11±0.00$^{a}$ | 0.001±0.00$^{b}$ | 0.23±0.02$^{a}$ | 0.68±0.06$^{c}$ | 1.03±0.18$^{b}$ | 0.08±0.06$^{ab}$ | 0.44±0.05$^{b}$ | 0.21±0.01$^{bc}$ | 1.84±0.04$^{b}$ | 0.08±0.02$^{b}$ | 0.23±0.00$^{ac}$ |
| U4 | 7.54±0.90$^{b}$ | 8.62±0.69$^{c}$ | 0.11±0.01$^{a}$ | 0.30±0.02$^{b}$ | 0.06±0.00$^{a}$ | 0.002±0.00$^{a}$ | 0.19±0.01$^{a}$ | 0.53±0.06$^{c}$ | 0.34±0.20$^{c}$ | 0.05±0.04$^{b}$ | 0.08±0.04$^{c}$ | 0.22±0.01$^{c}$ | 1.03±0.05$^{c}$ | 0.02±0.02$^{b}$ | 0.16±0.01$^{bc}$ |
| U5 | 5.05±0.32$^{c}$ | 5.68±0.31$^{d}$ | 2.31±0.18$^{b}$ | 0.39±0.02$^{c}$ | 0.19±0.01$^{b}$ | 0.001±0.00$^{b}$ | 2.75±0.20$^{b}$ | 0.05±0.00$^{b}$ | 0.06±0.03$^{d}$ | 0.09±0.03$^{ab}$ | 0.72±0.04$^{d}$ | 0.11±0.00$^{a}$ | 6.93±0.44$^{d}$ | 0.004±0.00$^{b}$ | 0.37±0.02$^{d}$ |

**Table S2.** Significant ($p < 0.05$) correlations between chemical-physical parameters and fungal ASVs calculated by Pearson coefficient among fungal ASVs at genus level (abundance > 2%) and chemical and physical parameters and highlighted in the Pearson correlations analysis (see Fig 6). TOC = Total Organic Carbon; TON = Total Organic Nitrogen; EC = Electrical Conductivity; Ca = calcium; Cl = chlorine; Cu = copper; Fe = iron; K = potassium; Li = lithium; Mg = magnesium; Mn = manganese; Mo = molybdenum; Na = sodium; P = phosphorous; S = sulphur; Sr = strontium; Ti = titanium; Zn = zinc.

| **row** | ***column*** | **cor** | **p** |
|---|---|---|---|
| Ca | *Glaciozyma* | 0.905761 | 1.15E-09 |
| S | *Glaciozyma* | 0.904775 | 1.29E-09 |
| Sr | *Glaciozyma* | 0.890068 | 5.83E-09 |
| *Mrakia* | *Vishniacozyma* | 0.803277 | 2.29E-06 |
| Mg | *Glaciozyma* | 0.793257 | 3.77E-06 |
| *Cladosporium* | *Mrakia* | 0.730821 | 5.00E-05 |
| *Cladosporium* | *Vishniacozyma* | 0.709213 | 0.000104 |
| *Malassezia* | *Vishniacozyma* | 0.677086 | 0.000279 |
| *Malassezia* | *Pichia* | 0.652117 | 0.000554 |
| *Leucosporidium* | *Sporobolomyces* | 0.648878 | 0.000603 |
| *Malassezia* | *Mrakia* | 0.643306 | 0.000696 |
| *Candida* | *Tausonia* | 0.607893 | 0.001627 |
| *Cladosporium* | *Malassezia* | 0.601644 | 0.00187 |
| depth | *Glaciozyma* | 0.594716 | 0.002176 |
| *Debaryomyces* | *Tausonia* | 0.587216 | 0.002554 |
| *Candida* | *Debaryomyces* | 0.586557 | 0.00259 |
| *Candida* | *Curvibasidium* | 0.583622 | 0.002755 |
| K | *Glaciozyma* | 0.574536 | 0.003321 |
| EC | *Glaciozyma* | 0.552255 | 0.005139 |
| TON | *Mrakia* | 0.523999 | 0.008583 |
| *Leucosporidium* | *Naganishia* | 0.486825 | 0.015843 |
| *Nakazawaea* | *Vishniacozyma* | 0.478326 | 0.018063 |

| | | | |
|---|---|---|---|
| Ti | *Vishniacozyma* | 0.465358 | 0.021932 |
| Fe | *Vishniacozyma* | 0.465339 | 0.021939 |
| Mo | *Malassezia* | 0.455723 | 0.02522 |
| *Curvibasidium* | *Naganishia* | 0.443955 | 0.029762 |
| Ti | *Malassezia* | 0.428543 | 0.036674 |
| Mn | *Vishniacozyma* | 0.426934 | 0.037463 |
| Cl | *Debaryomyces* | 0.424651 | 0.038607 |
| Fe | *Malassezia* | 0.422589 | 0.039662 |
| Mo | *Pichia* | 0.421298 | 0.040334 |
| Cu | *Malassezia* | 0.413147 | 0.04479 |
| Zn | *Vishniacozyma* | 0.412489 | 0.045166 |
| Zn | *Malassezia* | 0.406341 | 0.048798 |
| P | *Glaciozyma* | -0.44336 | 0.030008 |
| S | *Candida* | -0.47229 | 0.019788 |
| Ca | *Candida* | -0.51411 | 0.010169 |
| Sr | *Candida* | -0.52221 | 0.008853 |
| Mg | *Candida* | -0.52878 | 0.007894 |
| *Candida* | *Glaciozyma* | -0.54121 | 0.006313 |
